# Testing a TheoRY-inspired MEssage ('TRY-ME'): a sub-trial within the Ontario Printed Educational Message (OPEM) trial

**DOI:** 10.1186/1748-5908-2-39

**Published:** 2007-11-26

**Authors:** Jillian J Francis, Jeremy M Grimshaw, Merrick Zwarenstein, Martin P Eccles, Susan Shiller, Gaston Godin, Marie Johnston, Keith O'Rourke, Justin Presseau, Jacqueline Tetroe

**Affiliations:** 1 Health Services Research Unit, University of Aberdeen, Aberdeen, UK; 2 Clinical Epidemiology Program, Ottawa Health Research Institute, Ottawa, Canada; 3 Institute of Population Health, University of Ottawa, Ottawa, Canada; 4 Institute of Clinical Evaluative Sciences, Toronto, Ontario, Canada; 5 Department of Health Policy Management and Evaluation, University of Toronto, Toronto, Canada; Clinical Epidemiology Unit, Center for Health Services Sciences, Sunnybrook Hospital, Toronto, Ontario; Keenan Research Center, Li Ka Shing Knowledge Institute, St Michaels Hospital, Toronto, Ontario; 6 Institute of Health and Society, University of Newcastle upon Tyne, Newcastle upon Tyne, UK; 7 School of Nursing, University of Laval, Quebec City, Canada; 8 Department of Psychology, University of Aberdeen, Aberdeen, UK; 9 Department of Epidemiology and Community Medicine, University of Ottawa, Canada; 10 School of Psychology, University of Ottawa, Ottawa, Canada; 11 Knowledge Translation Branch, Canadian Institutes of Health Research, 160 Elgin Street, Ottawa, Canada

## Abstract

**Background:**

A challenge for implementation researchers is to develop principles that could generate testable hypotheses that apply across a range of clinical contexts, thus leading to generalisability of findings. Such principles may be provided by systematically developed theories. The opportunity has arisen to test some of these theoretical principles in the Ontario Printed Educational Materials (OPEM) trial by conducting a sub-trial within the existing trial structure. OPEM is a large factorial cluster-randomised trial evaluating the effects of short directive and long discursive educational messages embedded into ***informed***, an evidence-based newsletter produced in Canada by the Institute for Clinical Evaluative Sciences (ICES) and mailed to all primary care physicians in Ontario. The content of educational messages in the sub-trial will be constructed using both standard methods and methods inspired by psychological theory. The aim of this study is to test the effectiveness of the TheoRY-inspired MEssage ('TRY-ME') compared with the 'standard' message in changing prescribing behaviour.

**Methods:**

The OPEM trial participants randomised to receive the short directive message attached to the outside of ***informed ***(an 'outsert') will be sub-randomised to receive either a standard message or a message informed by the theory of planned behaviour (TPB) using a two (long insert or no insert) by three (theory-based outsert or standard outsert or no outsert) design. The messages will relate to prescription of thiazide diuretics as first line drug treatment for hypertension (described in the accompanying protocol, "The Ontario Printed Educational Materials trial"). The short messages will be developed independently by two research teams.

The primary outcome is prescription of thiazide diuretics, measured by routinely collected data available within ICES. The study is designed to answer the question, is there any difference in guideline adherence (i.e., thiazide prescription rates) between physicians in the six groups? A process evaluation survey instrument based on the TPB will be administered pre- and post-intervention (described in the accompanying protocol, "Looking inside the black box"). The second research question concerns processes that may underlie observed differences in prescribing behaviour. We expect that effects of the messages on prescribing behaviour will be mediated through changes in physicians' cognitions.

**Trial registration number:**

Current controlled trial ISRCTN72772651

## Background

In the clinical and health services, the problem of a knowledge-practice gap and its significant adverse effects on health and social welfare is increasingly being recognised and addressed [1,2]. However the effectiveness of interventions to translate knowledge into practice appears to vary across different clinical problems, contexts and organizations [3]. Current quantitative evaluations of professional behaviour-change strategies provide little insight into how interventions lead to behaviour change, and how they are moderated by different barriers and enablers to implementing evidence-based care [4]. This limits the ability to generalise from the findings of individual studies to other clinical problems and contexts. One of the challenges for implementation researchers is to develop general principles that could produce testable hypotheses about professional behaviour change that apply across a range of clinical contexts, thus leading to greater generalisability of research findings. Such principles may be provided by systematically developed and rigorously tested theories. The opportunity has arisen to test some of these theoretical principles in the context of the Ontario Printed Educational Materials (OPEM) Trial. The content of educational messages in this sub-trial will be constructed using both standard methods and methods inspired by theory.

### The Ontario Printed Educational Materials (OPEM) Trial

The OPEM trial [5] was originally designed to be a large, simple two (short, directive message or not) by two (long, discursive message or not) factorial cluster randomised trial, with participants randomised to one of four groups (control, short directive messages only, long discursive messages only, and both short and long messages). The messages are embedded in the ***informed ***newsletter, a free quarterly publication produced in Canada by ICES. The newsletter is a well-regarded evidence-based practice synopsis mailed quarterly since 1994 to 9,825 subscribers in Ontario, including all primary care physicians (except approximately 20 who opted to be removed from the mailing list). The short directive educational messages are produced on a postcard-sized card stapled to the outside of ***informed***. The long educational messages are produced as a one-page, two-sided insert into ***informed ***(indistinguishable from the rest of the periodical in size, style and editing), excluding the directive statements and including more background, an evidence-based guideline, and references. OPEM involves three replicated randomised trials in three successive editions of ***informed ***for three clinical behaviours (assertive hypertension and cholesterol treatment in diabetic patients; regular diabetic retinopathy screening; and use of thiazide diuretics in the initial management of hypertension). The TRY-ME study is designed to investigate the effects of theory-inspired and standard-construction short messages in the third replicate of these trials (use of thiazide diuretics in the initial management of hypertension). Routinely collected administrative data (OHIP, ODB and CIHI data) available within ICES will be used to measure changes in prescribing behaviour.

Guidelines for the initial treatment of hypertension (HT) among the elderly population originally recommended diuretics followed by beta-blockers as first line agents [6]. Beta-blockers are inferior in reducing long-term cardiovascular morbidity and mortality in elderly patients [7], while calcium channel blockers and ACE inhibitors appeared particularly useful in this group [8-10]. New guidelines repositioned diuretics as first-line agents, with calcium channel blockers and ACE inhibitors as second-line treatments for elderly patients with uncomplicated HT [11]. The recent publication of ALLHAT, a multi-centre randomized, double-blind clinical trial, has supported this choice by demonstrating that thiazide diuretics are superior to other antihypertensive agents in reducing cardiovascular disease outcomes [12].

In a separately funded, theory-based process evaluation study alongside these trials [13], we have developed theory-based questionnaire measures, to be administered pre- and post-intervention. The aim of the process study is to test for potential mediators of behaviour change so that mechanisms underlying trial effects can be identified. The OPEM process evaluation for the third trial (concerning thiazide prescription) will be used to address the second research question of the TRY-ME study.

The dissemination of printed educational materials (for example, mailing summaries of research findings to health care professionals) is a knowledge translation strategy commonly used in healthcare. There have been few evaluations of the effectiveness of this strategy [14,15], and little examination of the effect of message content on message effectiveness. Perhaps this arises from a lack of principles, categories, or dimensions by which to describe messages (see above). The proposed research offers one way of constructing and describing messages about evidence-based practice that is both testable and replicable.

### Theory-inspired messages

There are at least two plausible strategies for selecting a theoretical basis for this study. One is to choose from the well-researched principles relating to the effects of persuasive messages on attitude change, developed at Yale University in the 1950s [16]. Another is to select from theories that purport to predict actual behaviour.

Theories of persuasion propose that certain specified features of communication will be more persuasive (i.e., will more likely change attitudes) [17]. For example, one feature is credibility of the message source. When an audience perceives that the originator of the message is more credible (e.g., in this context, a scientist rather than a football player), the message will be more effective. The design of the OPEM trial already includes this principle, as the credibility of the ***informed ***newsletter is high (see above). Furthermore, the ICES and ***informed ***logos appear on the cards that present the short message, thus presumably enhancing credibility.

A further principle of persuasion theory, articulated in the Elaboration Likelihood model of persuasion [18-20], is the distinction between 'central' and 'peripheral' routes to persuasion. Individuals are more likely to be persuaded by an argument if they are able (i.e., motivated, have the skills and opportunity) to use the central route. This involves processing information deeply (e.g., by considering the arguments and carefully weighing up the positive and negative components). It is thus effective to present detailed information about the issue in question and include both sides of an argument. Individuals may still be persuaded if they are unable to engage in deep processing, but they will be influenced by factors that have nothing to do with the actual message content. In this peripheral route to persuasion, influence may occur through superficial cues such as the authority or attractiveness of the message source, or the amount of repetition of the message, a principle that drives strategies in the advertising industry. According to the Elaboration Likelihood model, the persuasive effect of a message that is processed through the peripheral route is weaker and more temporary than through the central route. On the reasonable assumption that primary care physicians are both capable and motivated to practice evidence-based care, the long message in the OPEM trial addresses this principle by presenting an elaborate message that can be processed using the central route. However, deep processing of information takes time and is a further demand on physicians, whose work is usually time-pressured and demanding. Hence the 'short message' arm of the OPEM trial attempts to provide relevant information formatted attractively with as little demand as possible on time and information processing resources. One highly generalisable aspect of the OPEM trial will relate to the three replications of the question of long versus short messages.

In summary, some aspects of persuasion theory are already represented in the OPEM trial. The theory-based process evaluation study, being conducted alongside the trial, includes a test of the predictions from this theory; namely, that exposure to the educational messages will change attitudes. Within this framework, the idea that altered attitudes will result in altered clinical actions is merely an assumption, albeit a plausible one, as persuasion theory is silent with respect to the effect of attitudes on behaviour.

**Figure 1:**
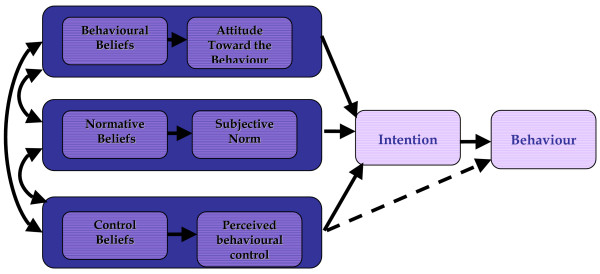
Theory of Planned Behaviour – Ajzen, 1991.

A number of theories propose a measurable link between attitudes and behaviour. This link has been empirically tested and appears to be robust. The TPB (Figure 1) is one such theory [21]. According to the TPB, the immediate precursor of behaviour is intention. To predict whether a person intends to do something, we need to know the person's attitude (i.e., whether the person is in favour of doing it). In addition, prediction will be improved by measuring two more variables: how much the person feels social pressure to do it ('subjective norm') and whether the person feels in control of the action in question ('perceived behavioural control'). Changing these three 'predictors' will increase the chance that the person will intend to do a desired action, and thus increase the chance of the person actually doing it. In a clinical consultation, the clinician's treatment decisions and actions are examples of intentional behaviour.

The TPB has been extensively tested in clinical settings, and includes guidance about how best to operationalise the constructs [22-24]. For example, the behaviour must be specified carefully in terms of the action itself (e.g., prescribing), its target (e.g., thiazide diuretics), the context (e.g., patients with hypertension) and the time (e.g., soon after diagnosis; in the near future). This is known as the TACT principle. The TRY-ME study will utilise the TPB because (a) it is well-tested in the clinical domain; and (b) specifications about operationalising the key variables are well developed [25-27].

The primary research question is whether a message inspired by the TPB will be more effective in changing clinical behaviour towards more evidence-based practice than a message based on 'standard' methods that are less informed by an explicit theoretical model. In addition (and as an indicator of underlying processes), we hypothesize that the variables in the theory that are represented in the theory-inspired message will be measurably improved among physicians who are exposed to theory-inspired message (compared with the standard message group and the control group), whereas the variables in the theory that are not represented in the theory-inspired message will not show such improvement.

For the TRY-ME study, the group randomised to receive the short directive message attached to the outside of the ***informed ***newsletter (the 'outsert') will be sub-randomised to receive either a standard message or a message informed by the TPB. The trial design will thus be expanded to a two (long message – 'insert' – or no insert) by three (theory-based outsert; standard outsert; or no outsert) design (see Table 1). The messages will relate to prescription of thiazide diuretics as first line drug treatment for hypertension (described in the accompanying protocol, 'The Ontario Printed Educational Materials trial') [5]. The short messages will be developed independently by two research teams, and the validity of the distinction between theory-inspired and standard messages will be established empirically.

**Table 1:** Design of replicate three of the OPEM Trial (the TRY-ME study)

**OPEM REPLICATE 3: Prescribing diuretics for first-line treatment of hypertension**			
		**LONG INSERT**	**NO INSERT**
**SHORT OUTSERT**	**Theory-based outsert**	1. Insert & theory- based outsert	2. Theory-based outsert only
	**Standard outsert**	3. Insert & standard outsert	4. Standard outsert only

**NO OUTSERT**		5. Insert Only	6. No printed educational message

The primary outcome is prescription of thiazide diuretics, measured by routinely collected data available within ICES. The study is designed to answer the question, is there any difference in guideline adherence (i.e., thiazide prescription rates) between physicians in these 6 groups?

### Aims and objectives

The aim of this study is to test the effectiveness of a theory-inspired short message compared with a 'standard' short message (without an explicit theoretical basis) and a long, discursive message in changing prescribing behaviour. The objectives are:

1. To develop two brief educational messages recommending the use of thiazide diuretics for the first line drug treatment of hypertension: one inspired by theory and one 'standard' (Phase I).

2. To test the effect of type of message (the two short messages and the long message) on frequency of prescription of thiazide diuretics using the two by three design described above. (Phase II). Prescribing behaviour will be measured two months before and six months after the intervention.

3. To use the theory-based process evaluation study to test the effects of these three messages in terms of the variables represented in the TPB (Phase III).

4. To test an explanatory proposition: that intervention effects will be mediated by changes in attitudes, subjective norms and intentions (Phase IV).

## Methods

### Ethics approval

The TRY-ME project has received ethics approval from the Research Ethics Board, Sunnybrook Campus, University of Toronto (Project identification number 135-2004).

### Study participants and setting

Participants are the primary care physicians in Ontario who receive the ***informed ***newsletter (and are thus participants in the third replicate of the OPEM trial) [5]. Data from this sample will be used to test the first hypothesis (specified in study objective two).

The subsample of these participants who will receive the TPB questionnaire will form the sample for the process study (relating to study objectives three and four). The questionnaire will be sent to 504 physicians randomly selected from the trial sample by the ICES-based investigators. The survey will be mailed to this subsample two months before and six months after the dissemination of the index edition of ***informed*.**

### Development of intervention materials

Two research teams will independently develop the wording of the two short messages. One team will consist of psychologists with experience in implementation research and clinical researchers experienced in the use of psychological theories (who will develop the theory-inspired message); the other team will consist of clinical researchers experienced implementation research and in the development of short educational messages directed to clinicians (who will develop the standard message). Each message will be designed to include the following:

• Banner

• Up to four bullet points

• Up to 85 words

• Key clinical messages with footnotes on back of card

• Cite the ALLHAT trial as the evidence base for the recommended behaviour

Following agreement on message wording, a graphic design consultant will format the messages using similar styles, font sizes and colours.

### Testing the validity of the intended distinction between theory-inspired and standard messages

The Aberdeen Health Psychology group, approximately 15 doctoral fellows, post-doctoral fellows and academics at the University of Aberdeen, who are not familiar with the OPEM study or with the way the two messages have been constructed, will be given the two messages and asked to make the following judgements with respect to each:

• What is the target behaviour?

• How clearly does the message specify the behaviour?

• Which theoretical constructs are reflected in this message?

Response formats will include confidence or extent ratings to produce continuous scales for appropriate analysis of the data. Order of presentation of the two messages will be counter-balanced, so that half the group will be presented with the theory-inspired message first.

Paired sample t-tests will be used to test differences between judgements. Validity check materials are presented in Additional file 1. The results will provide evidence regarding whether the 'theory-inspired' message has a greater amount of theoretical content (including the clarity of specification of the target behaviour) than the standard message.

### Development of survey instruments

These have been developed in accordance with the OPEM theory-based process evaluation study protocol [13]. Based on the TPB (Figure 1), a questionnaire will be developed to assess attitudes to prescription of thiazide diuretics as first line drug treatment of hypertension; perceived social pressure ('subjective norms') with respect to prescription of thiazide diuretics as first line drug treatment of hypertension; perceived control over this behaviour; and intention to prescribe thiazide diuretics as first line drug treatment of hypertension.

### Outcome and process variables

#### Primary outcome

Routinely collected data available within ICES will be used to measure changes in prescription of thiazide diuretics as the first line drug treatment for people with newly diagnosed hypertension. This will enable us to test for group differences by comparing the thiazide prescription rates of the groups exposed to the theory-inspired short message, the standard short message and the long message.

#### Process measures

The process evaluation survey instrument based on the TPB, to be administered pre- and post-intervention, will include measures of attitude, subjective norm, perceived behavioural control and intention.

### Sample size considerations

#### Primary outcome

Because the ***informed ***newsletter is mailed to over 9,000 primary care physicians, the sample size is adequate to both accommodate a second version of the short message and provide adequate numbers to recruit the required subsample for the process evaluation. Please see the OPEM trial protocol for additional details [5].

#### Process measures

Assuming a 50% response rate for each survey (pre- and post-intervention), we will mail the survey to 252 physicians in each of the six groups to achieve the sample size needed to have 80% power of detecting an effect size of 0.5 standard deviations using a significance level of 5%. Please see the OPEM process evaluation protocol for additional details [13].

#### Planned analyses

First, we will compare groups using methods appropriate for comparing independent samples (t-tests to compare two groups; analysis of covariance to compare groups adjusting for differences in baseline performance) to determine whether there have been changes in the prescription of thiazide diuretics across the study groups as hypothesised.

Second, in line with the protocol for the theory-based process evaluation study related to OPEM, we will test internal reliability of the questionnaire measures using Cronbach's alpha. If internal consistency is <0.7, we will explore whether we can improve this by omitting any individual item. We will then use a two-way Analysis of Covariance to test for group differences in scores for attitudes, subjective norms and intentions.

To test the mediation hypothesis, we will use a series of regression analyses (in the manner described by Baron and Kenny [28]) to explore the relationships between predictor variables (attitude and subjective norm), mediator (intention) and the dependent variable (recorded behaviour). If the dependent variable is markedly skewed, we will use generalized linear modelling regression to allow for this.

## Discussion

The benefits of theory-based interventions have been argued elsewhere [29]. Briefly, results of intervention studies that have a strong theoretical basis are potentially more generalisable than their atheoretical counterparts – or at least the limits of generalisability can be more easily specified. As theories identify process variables (in this case, the constructs that are proposed to mediate behaviour change), and how to operationalise them, the processes underlying change can be made explicit and investigated appropriately. This approach is thus likely to result in a cumulative science of implementation of evidence-based health care. In addition, the TRY-ME study enables us to distinguish between the content of an intervention and its mode of delivery. The same information (content) can be delivered using a variety of modes (e.g., educational group sessions; opinion leaders; printed materials), so holding the mode of delivery constant, as we have done in this study, enables us to investigate the question of content without contamination from the potential effects of different modes of delivery. Hence, this study will add to the body of knowledge that distinguishes between content and delivery mode.

Furthermore, an educational message is essentially a complex intervention. Theorizing of the intervention content also allows us to distinguish between intervention components (e.g., aspects of a message that focus on attitudes versus aspects that focus on subjective norms). By evaluating the effect of the intervention on each process variable, we can potentially identify which components are the active ingredients of the intervention. This work can thus lead to further studies that make explicit predictions about the effects of intervention components.

There are, however, limitations to this study that are dictated by pragmatic issues. For example, in experimental cognitive psychology, two sets of stimuli presented in a study like this would be exactly matched for word length and word frequency (i.e., how often each word is encountered in the daily use of a language, a proxy measure of word familiarity). Although the TRY-ME materials will be devised using a common set of criteria (see above), we will be subject to permissions and opinions of the editors of ***informed ***and this may limit our capacity to render the two versions of the insert comparable in this strict sense.

Furthermore, as argued above, the 'standard' method of developing the short message is not entirely devoid of theory; the underlying theory is merely less explicit for the standard message than for the theory-based message. The validation procedure for distinguishing between theory-based and standard messages will provide quantification of this possibility and is potentially a useful methodological component of this work.

In conclusion, the TRY-ME study will use theories and methods from psychology to devise a brief educational message in an attempt to change the behaviour of family physicians. We predict that the three kinds of messages (long; short and devised using standard methods; short and theory-based) will have different effects on behaviour change.

## List of abbreviations

CIHI – Canadian Institute for Health Information

ICES – Institute for Clinical Evaluative Sciences

ODB – Ontario Drug Benefit Program

OHIP – Ontario Health Insurance Plan

OPEM – Ontario Printed Educational Material

PEM – Printed Educational Material

TACT – Target, Action, Context, Time

TPB – Theory of Planned Behaviour

## Competing interests

The author(s) declare that they have no competing interests.

## Authors' contributions

JF contributed to the development of the theory-based message, designed the validation test for the theory-based versus non-theory-based messages, was a Co-I for the process evaluation study, and drafted the manuscript. JG contributed to the development of the theory-based message, was PI on the process evaluation study and a Co-I on the OPEM trial; MZ conceived the study, contributed to the development of the non-theory-based message, was PI for the OPEM trial and Co-I for the process evaluation study; ME contributed to the development of the theory-based message and was a Co-I on the process evaluation study; SS contributed to the development of the non-theory-based message; GG and MJ contributed to the development of the theory-based message and were Co-Is for the process evaluation study; KOR led on the analysis plan and sample size calculations; JP and JT contributed to the study design, analysis plans and draft manuscript. All authors commented on the design of the TRY-ME sub-trial and on drafts of the manuscript, and all approved the final version.

## Supplementary Material

Additional File 1 Validity check materials for TRY-ME (within the OPEM trial). The materials to be distributed to members of the Aberdeen Health Psychology Group to test whether the 'theory-inspired' message is judged to have a greater amount of theoretical content than the standard message. Click here for file
