# Supplementary material for: Testing a TheoRY-inspired MEssage ('TRY-ME'): a sub-trial within the Ontario Printed Educational Message (OPEM) trial
Source: Implement Sci. 2007 Nov 26;2:39. doi: 10.1186/1748-5908-2-39 (PMC2216024; doi:10.1186/1748-5908-2-39)
Supplement: Additional File 1 — Validity check materials for TRY-ME (within the OPEM trial). The materials to be distributed to members of the Aberdeen Health Psychology Group to test whether the 'theory-inspired' message is judged to have a greater amount of theoretical content than the standard message. [file 1748-5908-2-39-S1.doc]

***Testing a theory-inspired message: Validity check materials***

##### JUDGEMENT TASK

You will be asked to read two brief messages. General medical practitioners in Canada will be sent one of these messages, with the aim of influencing their clinical behaviour. We will ask you to answer two questions about each message.

Please read the messages and answer the questions in the order in which they appear.

MESSAGE 1: [Insert wording of non theory-informed message]

1a. From the wording presented above, what is the target behaviour? (ie the behaviour that GPs are being asked to enact). Please specify in as much detail as possible.

1b. How clearly does the message specify the behaviour?

(Please mark one of these seven spaces with a cross **X**)

Not at all ­­_____ _____ _____ _____ _____ _____ _____ Extremely

clearly clearly

2a. Which theoretical constructs are reflected in this message?

Please list one construct on each of the rows in the table below, and then indicate how certain you are about your decision by marking one of the seven spaces with a cross **X**. If you need more rows, please enter further details over the page.

If you feel that it does not reflect any constructs, please mark the box below with a cross **X**.

| **Construct** | **Rank order (impor-tance)** |  | **Certainty ratings**  **(please mark with a X)** | | | | | | |  | |
| --- | --- | --- | --- | --- | --- | --- | --- | --- | --- | --- | --- |
|  |  | Extremely uncertain | __ | __ | __ | __ | __ | __ | __ | | Extremely certain |
|  |  | Extremely uncertain | __ | __ | __ | __ | __ | __ | __ | | Extremely certain |
|  |  | Extremely uncertain | __ | __ | __ | __ | __ | __ | __ | | Extremely certain |
|  |  | Extremely uncertain | __ | __ | __ | __ | __ | __ | __ | | Extremely certain |
|  |  | Extremely uncertain | __ | __ | __ | __ | __ | __ | __ | | Extremely certain |

□ This messages reflects NO theoretical constructs

2b. Finally, how important does each construct appear to be in this message? Please write a number in the second column of the table above, to indicate the rank order of the constructs, from the most important construct (1).

MESSAGE 2: [Insert wording of theory-informed message]

1a. From the wording presented above, what is the target behaviour? (ie the behaviour that GPs are being asked to enact). Please specify in as much detail as possible.

1b. How clearly does the message specify the behaviour?

(Please mark one of these seven spaces with a cross **X**)

Not at all ­­_____ _____ _____ _____ _____ _____ _____ Extremely

clearly clearly

2a. Which theoretical constructs are reflected in this message?

Please list one construct on each of the rows in the table below, and then indicate how certain you are about your decision by marking one of the seven spaces with a cross **X**. If you need more rows, please enter further details over the page.

If you feel that it does not reflect any constructs, please mark the box below with a cross **X**.

| **Construct** | **Rank order (impor-tance)** |  | **Certainty ratings**  **(please mark with a X)** | | | | | | |  | |
| --- | --- | --- | --- | --- | --- | --- | --- | --- | --- | --- | --- |
|  |  | Extremely uncertain | __ | __ | __ | __ | __ | __ | __ | | Extremely certain |
|  |  | Extremely uncertain | __ | __ | __ | __ | __ | __ | __ | | Extremely certain |
|  |  | Extremely uncertain | __ | __ | __ | __ | __ | __ | __ | | Extremely certain |
|  |  | Extremely uncertain | __ | __ | __ | __ | __ | __ | __ | | Extremely certain |
|  |  | Extremely uncertain | __ | __ | __ | __ | __ | __ | __ | | Extremely certain |

□ This messages reflects NO theoretical constructs

2b. Finally, how important does each construct appear to be in this message? Please write a number in the second column of the table above, to indicate the rank order of the constructs, from the most important construct (1).

**THANK YOU FOR YOUR TIME AND EXPERTISE. A FULL EXPLANATION AND DETAILS OF THIS STUDY WILL BE PROVIDED LATER IN THIS SESSION**.
